# 

**Published:** 1914-07-11

**Authors:** 


### Appointments.

Mr. A. H. Bostock, M.R.C.S., L.R.C.P., of South Street, Chichester, has been elected honorary surgeon of the Royal West Sussex Hospital, Chichester, in place of Mr. F. Skaife, resigned.

Mr. E. Allan, fifth assistant medical officer at Hanwell Mental Hospital, has resigned his appointment, and Mr. J. T. H. Madill, the sixth assistant medical officer, has been promoted to take his place.

The Wandsworth Board of Guardians have decided, subject to the approval of the Local Government Board, to grant Dr. E. F. W. Nixey, deputy medical superintendent of St. John's Hill Infirmary, £50 per annum as an allowance for rations. This will bring his salary up to £350 a year, the same as his predecessor received.

Dr. J. Edmond, third assistant medical officer at the Lambeth Infirmary, has been appointed medical officer at the Pay Ward at Guy's Hospital.

Dr. Harry M. Grey, M.R.C.S., L.R.C.P. (Lond.), has been appointed by the Camberwell Board of Guardians assistant medical officer at their children's homes. He is at present *locum tenens* at Camberwell Infirmary, but was previously assistant in the electricity department at the Middlesex Hospital, senior house surgeon at Walsall and District Hospital, and senior resident assistant medical officer at the workhouse infirmary, Portsmouth.

RATES OF SUBSCRIPTION: Twelve months, 6/6; Six months, 4/-; Three months, 2/-, post free to any address in the United Kingdom. Abroad, Twelve months, 8/8; Six months, 5/-; Three months, 2/6, post free.—Address The Subscription Dept., "THE HOSPITAL," The Hospital Building, 28/29 Southampton Street Strand, London W.C.

